# Resource landscapes explain contrasting patterns of aggregation and site fidelity by red knots at two wintering sites

**DOI:** 10.1186/s40462-018-0142-4

**Published:** 2018-12-20

**Authors:** Thomas Oudman, Theunis Piersma, Mohamed V. Ahmedou Salem, Marieke E. Feis, Anne Dekinga, Sander Holthuijsen, Job ten Horn, Jan A. van Gils, Allert I. Bijleveld

**Affiliations:** 1 NIOZ Royal Netherlands Institute for Sea Research, Department of Coastal Systems, and Utrecht University, P.O. Box 59, 1790 AB Den Burg, Texel The Netherlands; 2 Centre for Biological Diversity, School of Biology, University of St Andrews, Fife, KY16 9TF UK; 3 Rudi Drent Chair in Global Flyway Ecology, Conservation Ecology Group, Groningen Institute for Evolutionary Life Sciences (GELIFES), University of Groningen, PO Box 11103, 9700 CC Groningen, The Netherlands; 4 EBIOME Ecobiologie Marine et Environnement, Département de Biologie, L’université de Nouakchott Al-Aasriya, BP. 880 Nouakchott, Mauritania; 5 Present Address: Sorbonne Université, CNRS, Station Biologique de Roscoff, Laboratoire Adaptation et Diversité en Milieu Marin, UMR 7144, CS90074, 29688 Roscoff Cedex, France

**Keywords:** Aggregation, Foraging, Heterogeneity, Intertidal, Movement, Predator-prey interactions, Red knots, Site fidelity, Time-of-arrival, Tracking

## Abstract

**Background:**

Space use strategies by foraging animals are often considered to be species-specific. However, similarity between conspecific strategies may also result from similar resource environments. Here, we revisit classic predictions of the relationships between the resource distribution and foragers’ space use by tracking free-living foragers of a single species in two contrasting resource landscapes. At two main non-breeding areas along the East-Atlantic flyway (Wadden Sea, The Netherlands and Banc d’Arguin, Mauritania), we mapped prey distributions and derived resource landscapes in terms of the predicted intake rate of red knots (*Calidris canutus*), migratory molluscivore shorebirds. We tracked the foraging paths of 13 and 38 individual red knots at intervals of 1 s over two and five weeks in the Wadden Sea and at Banc d’Arguin, respectively. Mediated by competition for resources, we expected aggregation to be strong and site fidelity weak in an environment with large resource patches. The opposite was expected for small resource patches, but only if local resource abundances were high.

**Results:**

Compared with Banc d’Arguin**,** resource patches in the Wadden Sea were larger and the maximum local resource abundance was higher. However, because of constraints set by digestive capacity, the average potential intake rates by red knots were similar at the two study sites. Space-use patterns differed as predicted from these differences in resource landscapes. Whereas foraging red knots in the Wadden Sea roamed the mudflats in high aggregation without site fidelity (i.e. *grouping nomads*), at Banc d’Arguin they showed less aggregation but were strongly site-faithful (i.e. *solitary residents)*.

**Conclusion:**

The space use pattern of red knots in the two study areas showed diametrically opposite patterns. These differences could be explained from the distribution of resources in the two areas. Our findings imply that intraspecific similarities in space use patterns represent responses to similar resource environments rather than species-specificity. To predict how environmental change affects space use, we need to understand the degree to which space-use strategies result from developmental plasticity and behavioural flexibility. This requires not only tracking foragers throughout their development, but also tracking their environment in sufficient spatial and temporal detail.

**Electronic supplementary material:**

The online version of this article (10.1186/s40462-018-0142-4) contains supplementary material, which is available to authorized users.

## Background

Outside of the reproductive period, the main reason for animals to move is to feed. In this situation, the distribution of resources across the environment is the main determinant of animal space use patterns [1]. Therefore, the fact that different populations of one species often have the same space use characteristics may not only be the consequence of similar physical and cognitive traits, but also of similar resource environments.

Resource environments can affect space use in several ways. Here, we distinguish between two types of space use patterns. First, the environment influences the degree of *aggregation*: spatial patterns in the locations of different animals across the landscape at a single point in time [2–5]. In that sense, aggregation can be quantified by a family of space use variables that include dispersion, group size, degree of sociality and inter-individual distances. Second, the environment also influences *site fidelity*: spatial patterns in the locations of single individuals over time [6–14]. In this sense, site fidelity includes space use measures such as dispersal, home range, return rate and exploration behaviour.

**Fig. 1:**
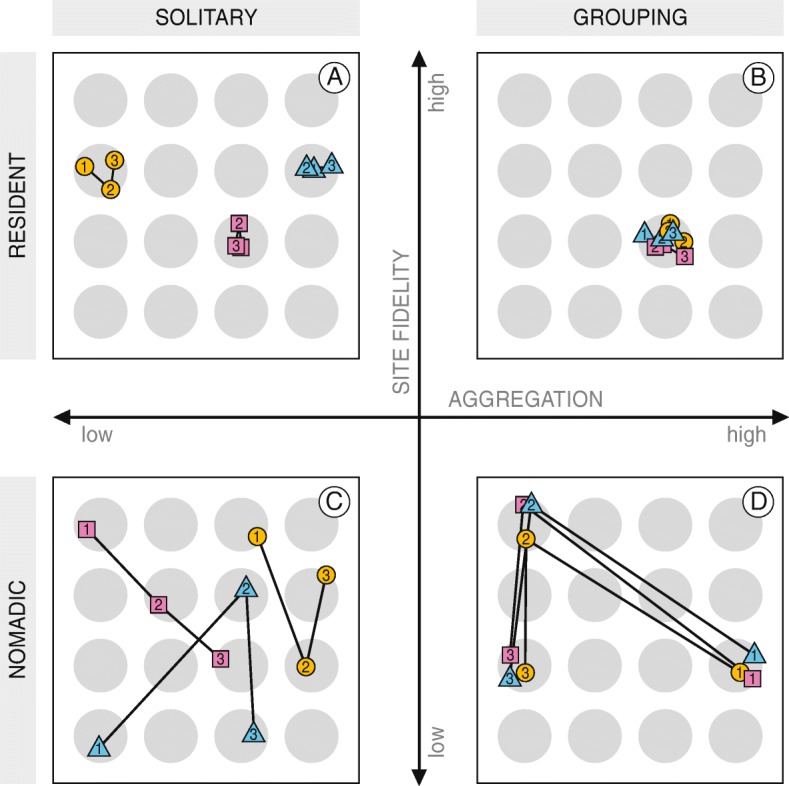
Four extreme patterns in population space use, with the degree of aggregation on the x-axis and the degree of site fidelity on the y-axis. For clarity, we named the four extremes *solitary residents* (**a**), *grouping residents* (**b**), *solitary nomads* (**c**) and *grouping nomads* (**d**). Any intermediate pattern is also possible. Grey circles represent resource patches. Symbols denote three different individuals, and the thin lines connect subsequent positions of the same individual for three time steps, which are denoted by the numbers in the symbols. Individual positions within patches are arbitrary

In theory, all combinations of aggregation and site fidelity are possible. For clarity, we plotted different space use patterns produced by foragers that choose between several discrete resource patches in a series of time steps (Fig. 1). The four panels represent the extreme cases, to which we refer as *solitary residents* (low aggregation and high site fidelity, Fig. 1a), *grouping residents* (high aggregation and high site fidelity, Fig. 1b), *solitary nomads* (low aggregation and low site fidelity, Fig. 1c) and *grouping nomads* (high aggregation and low site fidelity, Fig. 1d). Although all combinations are possible, different environments may favour different combinations of aggregation and site fidelity.

### Costs and benefits of aggregation and site fidelity in different environments

A high degree of aggregation allows for shared vigilance for predators and decreases per capita predation risk [15]. It also enables the transfer of social information [16], for example on foraging opportunities [17]. The main cost of grouping by foraging animals is competition for resources. Competition for resources happens when foragers decrease each other’s intake rate, which can result from resource depletion as well as from interference competition [18, 19]. Resource competition will be generally be reduced when local resource abundance is higher (less depletion) [20, 21] and when resource patch surface is larger (less interference) [19, 22]. The optimal degree of aggregation will thus depend both on absolute local resource density and on resource patch size, i.e. the scale of spatial heterogeneity.

Site fidelity can be beneficial because it reduces variance in foraging success, for example when foragers return to good patches and stay away from bad ones [6]. Given that the resource environment is correlated over time, foragers are expected to show high site fidelity to specific patches when the difference in quality between foraging locations is high [23]. When the resource environment is not (or even negatively) temporally correlated, the relation between spatial heterogeneity and site fidelity is expected to reverse [6]. Then, high site fidelity is only expected if the spatial differences in quality are so low that it does not pay to search the environment for better patches [6]. Site fidelity becomes particularly costly when foraging has a strongly negative effect on the availability of resources. This can happen through depletion [24] or through predator avoidance behaviour by the prey [15].

When foraging has a negative effect on the availability of resources, both the abundance of foragers in a patch and the foraging time spent at one patch are constrained by local resource density [2, 6]. For this reason, aggregation may negatively influence the optimal degree of site fidelity. A ‘*grouping resident*’ pattern of space use (Fig. 1b) may be expected only when local resource abundances are very high and competition is low or absent. If resources are less abundant, they may still occur in large patches and allow foragers to aggregate. In this case, however, effects of resource depletion will be strong and limit site fidelity, which results in a ‘*grouping nomad*’ pattern of space use (Fig. 1d). Territorial behaviour may counter the aggregation of conspecifics and allow high site fidelity, but only when patches are small and thus defendable [25–27]. As a result, a ‘*solitary resident*’ pattern of space use (Fig. 1a) is expected only when resources are distributed in small patches with high local resource abundances. When patches are small and contain so few resources that the prolonged presence of even one forager cannot be sustained, a ‘*solitary nomad*’ pattern of space use (Fig. 1c) may be the only option.

### Measuring aggregation, site fidelity, and the resource landscape in two contrasting environments

Considerable variation in space-use patterns has been described within single species in response to different resource distributions, both in terms of *aggregation* [28–30] as well as in terms of *site fidelity* [31, 32], but not simultaneously. In this study, we measured both aggregation and site fidelity at two different wintering sites of a migratory shorebird, the red knot (*Calidris canutus*). At both sites, the red knots forage on mollusc prey in superficially similar intertidal habitats that nonetheless contrast in the spatial distribution of molluscs. In both areas, with radio tags, we collected detailed foraging tracks of individual red knots simultaneously, and performed detailed and spatially explicit resource sampling.

Previous studies have shown that the resource intake rates by red knots in the two areas are often not limited by search time or handling time, but instead by constraints on digestion and sulphide detoxification rate [33–36]. To compare the resource landscapes between the two areas, we therefore constructed spatial maps of the estimated potential energy intake rate (ash-free dry flesh mass) using a functional response model that was tailored for red knots in these specific areas and considers all these constraints [33–36]. The model was fitted to the data from the resource-sampling scheme. In addition, we analysed the contents of red knot droppings in both areas and considered only those mollusc species that comprised more than 1 % of the estimated red knot’s diet in terms of ash-free flesh mass. This method requires extensive sampling efforts and lab work (see methods) and relies on detailed experimental studies of prey choice, but it results in much more explicit and precise estimates than indirect indices of resource abundance, such as chlorophyll indices derived from satellite images [37–39].

The resource landscapes will yield predictions on differences in space-use patterns of red knots between the two areas. By comparing these predictions with the observed levels of aggregation and site fidelity, we investigated whether the distribution of resources could indeed affect the patterns of space use by red knots. The statistical significance of differences in the patterns of aggregation and of site fidelity were tested by a repeated randomization procedure, randomizing the red knot identity and tide of each itinerary and counting the proportion of randomizations in which a more extreme pattern occurred than the observed pattern [40].

## Methods

### Tracking red knots

Data was collected in the two main wintering areas of red knots along the East-Atlantic Flyway: the Wadden Sea in The Netherlands (53°15’N, 5°15′E), and the Banc d’Arguin in Mauritania (19°53’N, 16°17’W) [41]. These two populations, belonging to the subspecies *C. c. islandica* and *C. c. canutus* respectively, are very similar in morphology and genetically barely distinct [42, 43]. The *canutus* knots stage in the Wadden Sea during north- and southward migration, where they likely occur in mixed flocks with *islandica* knots [41, 44] and cannot be told apart visually [44, 45]. In the Wadden Sea in August, daily temperatures were roughly the same as at Banc d’Arguin in January, and given the similarity in the tidal movements, so would be daily energy expenditures [46].

Using a novel automated tracking system with a high resolution both in space and time [47, 48], detailed tracks of individual red knots were collected in both areas. From a previously published study in the Dutch Wadden Sea [49], we used data from 13 red knots tracked between 12 and 26 August 2011. At Banc d’Arguin, 46 red knots were released with a tag, and tracked between 9 January and 13 February 2013 [50]. In the Wadden Sea we tracked the subspecies *C. c. islandica*, which spends the winter in intertidal systems in north-western Europe, including the Wadden Sea. At Banc d’Arguin, tracked red knots were of the subspecies *C. c. canutus*, which winters in in West-Africa, with a majority at Banc d’Arguin, and with some staging in the Dutch Wadden Sea in late summer when returning from the breeding grounds in Taimyr, north-central Siberia [44, 51].

The 6.5 g radio tags (ranging from 5.5 to 7.5 g; < 5% of body mass) were glued on the rump with Superglue [52]. Every second the tags emitted an individual-specific radio signal, to be received by an array of receiver stations in the study area. When received by at least three receiver stations, the tag’s location was calculated from arrival times at the different stations [48, 53] and stored in a database.

### From tracking data to foraging itineraries

During low tide, red knots move over intertidal mudflats in search of buried mollusc prey, which they find by repeated probing of the sediment with their ca. 3.5 cm long bill [54–56]. As the tide retreats, red knots fly to foraging locations, roughly 100 m to 10,000 m away from the roost, and visit one or several different locations before returning to the roost when the water returns [49, 50]. After landing, red knots search for prey on foot, and may walk 100 m or more between flights. Hence, red knots search for resources on two spatial scales. They move between foraging locations by flight, and move on foot within these locations. In accordance with this typical pattern of movement, individual foraging itineraries were described as a sequence of patch visits during a single low tide period [49]. To this end the raw position data were summarized into a series of residence patch visits during each low tide period (2 h before to 2 h after low tide). This was done in four steps. First, the raw position data was median-filtered using a 5-point sliding window. Then, using the method described by Barraquand & Benhamou [14], the duration of stay within 125 m of each position was calculated, reflecting half the distance between resource sampling stations as well as roughly the red knots’ scale of movement by flight. Excursions outside the radius for less than 30 s were allowed. The resulting sequence of “residence times” was segmented by the penalized contrasts method [57] into locations with an arrival and departure time. Finally, adjacent residence patches closer than 125 m were combined into one residence patch. Patch visits shorter than 10 min were not used in the analysis, because birds were then probably travelling rather than foraging [49]. The subsequent patch visits of a single bird during a single low tide period were defined as one foraging itinerary. This resulted in 144 foraging itineraries of 13 different birds in the Wadden Sea, and 1323 itineraries of 38 birds in the Banc d’Arguin. For further details we refer to Bijleveld et al. [49] for the Wadden Sea study and Oudman et al. [50] for the Banc d’Arguin study. The much lower number of itineraries in the Wadden Sea was due to technical issues that are inherent to the use of a prototype system, which concerned tag water-resistance and radio receiver software. These issues were resolved in the later study at Banc d’Arguin. Field observations in both study areas in subsequent years suggested that tagged red knots did not suffer from higher mortality than other colour-ringed red knots (TO, AIB and JtH, unpublished data).

### Measuring aggregation and site fidelity

**Fig. 2:**
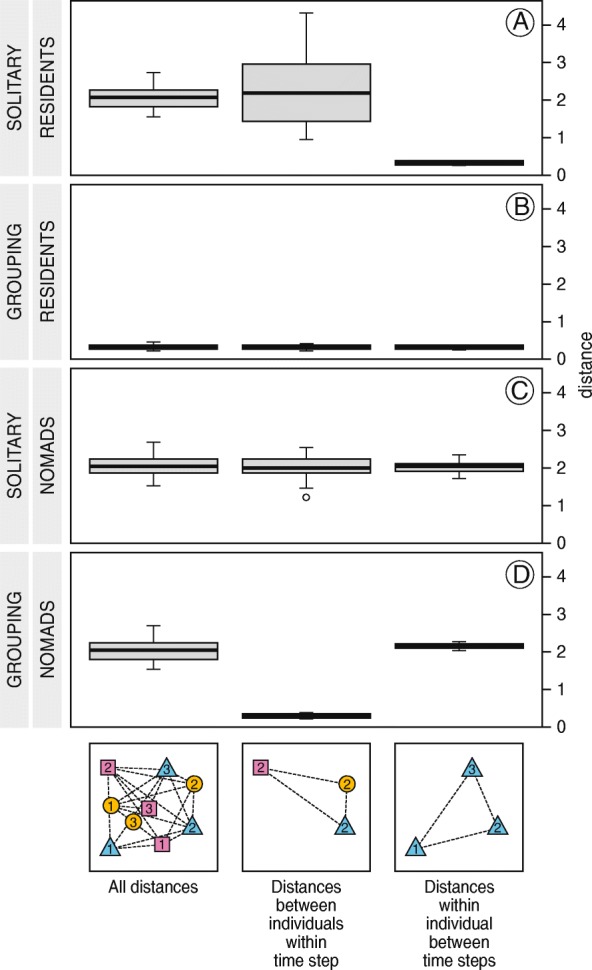
Mean distances between individuals in simulations of four different space use strategies. Each panel shows one of four extreme strategies in Fig. 1. Data was simulated for 16 individuals during 16 time steps. The distance between neighbouring patches is taken as the unit of distance. Left boxes show the distances between all combinations of locations, averaged per individual. Middle boxes show the distances between all locations and the other locations in the same time step, averaged per individual. Right boxes show the distances between all locations and the other locations of the same individual, averaged per individual

A common problem with absolute measures of aggregation (e.g. group size or distance to the nearest neighbour) from tracking data is that all individuals in the area must be tracked to derive an accurate estimate. We avoided this problem by assuming that the tagged birds mixed with the non-tagged birds (as field observations in both areas confirmed) and using a relative measure of aggregation. First, we computed the pairwise mean distances of each itinerary to all other itineraries, regardless by which individual and in which low tide period the itineraries had been made. That resulted in $\left(\genfrac{}{}{0pt}{}{144}{2}\right)=\mathrm{10,296}$mean distances in the Wadden Sea and $\left(\genfrac{}{}{0pt}{}{1323}{2}\right)=\mathrm{874,503}$ mean distances in Banc d’Arguin. Then, we calculated whether the mean distance between all pairs of itineraries in the *same* low tide period (middle boxes in Fig. 2) were smaller than the mean distance between all combinations of itineraries at *different* low tide periods (left boxes in Fig. 2). Similarly, we used a relative measure of site fidelity: we calculated whether the mean distances between all pairs of itineraries of the *same* individual (right boxes in Fig. 2) were smaller than the mean distances between all combinations of itineraries from *different* individuals (left boxes in Fig. 2) [40, 58]. Applying this method to the simulated data from the conceptual model (Fig. 1), shows that it allows for the identification of *solitary residents* (Fig. 2a) and *grouping nomads* (Fig. 2c). It also shows that whether animals are classified as *grouping residents* (Fig. 2b) or as *solitary nomads* (Fig. 2d) depends on the scale of observation; both cases show no difference between the non-randomized and the randomized distances.

To compute an absolute measure of the mean distance between two itineraries, the distance between them was calculated each 5 min from 2 h before to 2 h after low tide, as the distance between the patch location in the first itinerary to the nearest patch location in the second itinerary within one hour. These distances were averaged to obtain a single mean distance between the two itineraries. An earlier analysis of the Banc d’Arguin data showed that red knots moved less during the day than during the night [50, 59]. To avoid an effect of this temporal trend on the estimate of site fidelity, daytime locations were only compared with other daytime locations and night time locations were only compared with other locations at night. Locations recorded in the hour before sunrise and after sunset were not used at all. Mean distances between itineraries of different birds in the same low tide period (middle boxes in Fig. 2) were calculated by averaging for each itinerary the distances to all itineraries by other birds in the same tide. Likewise, mean distances between itineraries within birds in different low tide periods (right boxes in Fig. 2) were calculated by averaging, for each itinerary, the distances to all other itineraries by the same bird.

Due to the complex structure of the data, significance of the observed differences between the mean distances of all itineraries and the mean distances between individuals within tides (to show aggregation), as well as the differences between mean distances of all itineraries and the mean distances within individuals between tides (to show site fidelity), was tested by a repeated (10,000 times) randomization procedure [40, 60]. For each randomization, all tag-IDs and tide-IDs from the original itineraries were randomly re-assigned, the distances between all pairwise combinations of itineraries calculated and then averaged per itinerary, to arrive at a randomized estimate for the average distance between itineraries. To calculate a randomized estimate for its difference with between-individual distances within tides, tide-IDs were randomized per individual, all pairwise distances between itineraries within the same tide calculated, and then averaged per itinerary. To arrive at a randomized estimate for mean distances within individuals between tides, tag-IDs were randomized per tide, all pairwise distances between itineraries within the same tag calculated, and then averaged per itinerary [40]. Significance of the aggregation pattern and the site fidelity pattern was assessed in both areas independently by calculating the proportion of simulations that resulted in a more extreme difference than the actual observed difference (two-tailed *p*-value). To assess significance of differences between the Wadden Sea and Banc d’Arguin, mean distances were averaged per tag to arrive at independent observations, and tested by linear regression.

### Resource sampling

The mollusc food of red knots was sampled at 880 locations in the Wadden Sea between 15 and 19 July 2011, and at 265 locations at Banc d’Arguin between 4 and 17 January 2013. Both sampling schemes consisted of a 250 m grid with a spatial accuracy of approximately 10 m, with an additional 20% locations placed randomly on the grid lines [49, 61]. Samples were taken by pushing a core into the sediment to at least 20 cm depth, and sieving the top 4 cm over a 1 mm mesh (either one core with a surface of 1/56 m^2^ or two cores of 1/112 m^2^). All molluscs were collected and stored in 4% formaldehyde, except for bivalves longer than 8 mm in the Wadden Sea, which were frozen.

All individual molluscs were identified to the species level, and length was measured to the nearest 0.1 mm. Dry mass of the shell (DM_shell_) and ash-free dry mass of the flesh (AFDM_flesh_) was measured in a subset of the samples as described by Piersma et al. [62]. Individuals below 8 mm (Wadden Sea) and 5 mm (Banc d’Arguin) in length were pooled before weighing when in the same sample. In *Limecola balthica, Ensis directus* and *Mya arenaria*, flesh and shell were weighed together, and the ash-free dry mass of the shell was estimated with calibration lines from Zwarts [63]. The gastropod *Peringia ulvae* was also weighed as a whole, assuming that 12.5% of organic matter resided in the shell [64]. For bivalve species weighed whole, DM_shell_ and AFDM_flesh_ of the unweighted individuals were estimated by non-linear local regression of the log-transformed masses and lengths of the weighed individuals [65].

To determine which potential prey species contributed to the diet of red knots, we analyzed the composition of the droppings and calculated the relative contribution of different prey species to the diet [66, 67]. Droppings were collected in the field at locations where radio-tagged red knots were observed foraging (2–10 droppings at 32 locations in the Wadden Sea and 45 locations in Banc d’Arguin). The droppings were aggregated per location and sieved over a 300 μm mesh. All bivalve hinges and last coils of gastropods were identified to the species level and measured. Each measurement was converted to an estimated AFDM_flesh_ mass, using species-specific calibration measurements of whole individuals [66, 67].

### Calculating potential resource intake rates

Potential intake rates (mg AFDM_flesh_ per second) of the relevant mollusc species were estimated at each sampling station as a function of the observed densities. The estimates were calculated with an experimentally tested functional response model for red knots that forage on mollusc prey, taking into account search time and size- and species-dependent handling time and digestive quality of the prey. It also accounts for the fact that the main available prey species at Banc d’Arguin, *Loripes lucinalis*, contains high levels of sulphur and/or sulphur-compounds that limit the intake by red knots [34–36]. The model also takes into account that digestion rate by red knots varies among individual red knots [68] and scales to the square of gizzard mass [33, 69]. Gizzard masses were measured by ultrasonography [70, 71] immediately after the catch, and were lower in the Wadden Sea (mean ± SD, 7.0 ± 2.0 g) than at Banc d’Arguin (8.5 ± 1.8 g). Only those prey species that were estimated to comprise at least 1% of the red knots diet, in terms of AFDM_flesh_ were considered. A more detailed explanation of the functional response model and its parameterization is given in Additional file 1: Appendix 1.

### The distribution of resources

To determine the range of autocorrelation in the resource landscape, spatial autocorrelation in the predicted AFDM_flesh_ intake rates was calculated at discrete distances of 50 m with the function “correlog” in R-package “ncf” [72], using *Moran’s I* index as the measure of autocorrelation [49, 73, 74]. The autocorrelation range was estimated by the distance at which the spatial autocorrelation went below 0.1, which can be interpreted as a measure of resource patch size [73].

## Results

### Aggregation and site fidelity of tagged red knots

**Fig. 3:**
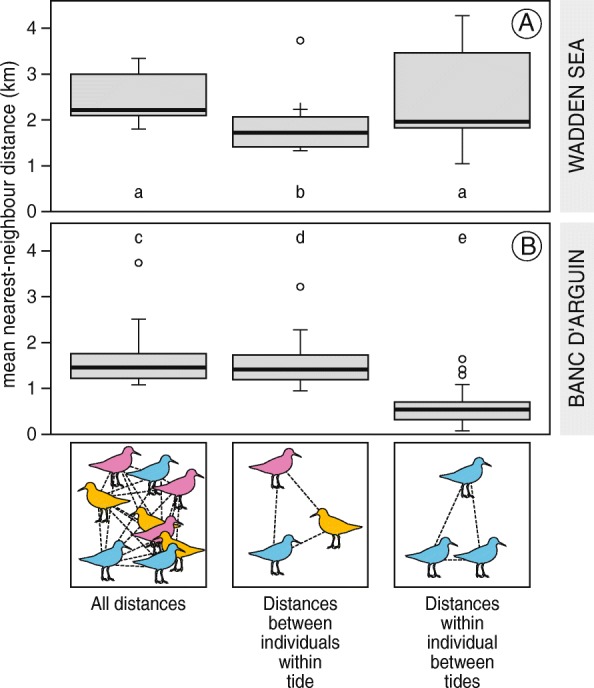
Aggregation and site fidelity of red knots in the Wadden Sea and at Banc d’Arguin. Shown are the mean distances between all itineraries (left), the mean distances between itineraries of the same bird in different low tide periods (middle, a measure of site fidelity), and mean nearest-neighbour distances between itineraries of different birds during the same low tide (right, a measure of aggregation). Data is averaged per bird. Boxes a, b, c, d and e differ significantly from each other. Significance was assessed by repeatedly (10,000 times) drawing a random sample from all mean distances shown in the left boxes

In the Wadden Sea, mean distances between red knot itineraries *in the same tide* were significantly smaller than the mean distances between *all* combinations of itineraries (on average 1900 m and 2500 m, *p* < 0.001, Fig. 3a), meaning that they aggregated in some part of the total foraging range during each low tide. The tagged red knots did not show site fidelity in the Wadden Sea, as mean distances between itineraries *of the same bird* were not significantly smaller than the mean distances between *all* combinations of itineraries (both 2500 m on average, *p* = 0.09, Fig. 3a). These differences agree with the differences in the simulated data of “grouping nomads” (Fig. 2d). Maps of red knot locations are provided as Additional file 1: Figure A2.

In contrast, red knots at Banc d’Arguin showed strong site fidelity, as mean distances between all combinations of itineraries *of the same bird* were much smaller than mean distances between *all* combinations of itineraries (on average 600 m and 1600 m, *p* < 0.01, Fig. 3b). Red knots at Banc d’Arguin also did aggregate, but the area used per tide was only slightly smaller than the area used by the study population. Distances between itineraries of birds in the same tide were on average 100 m smaller than distances between *all* combinations of itineraries (1500 m and 1600 m, *p* = 0.01, Fig. 3b). These differences agree best with the simulated data of “solitary residents” (Fig. 2a).

Note that all absolute distances averaged per bird were larger in the Wadden Sea than at Banc d’Arguin (2500 and 1600 m, F_1,49_ = 27.3, *p* < 0.001), suggesting that overall the tagged red knots used a larger area within the range covered by the receiver stations in the Wadden Sea than at Banc d’Arguin. Average distances between tagged birds within the same tide were also larger in the Wadden Sea than at Banc d’Arguin (1800 and 1500 m, F_1,49_ = 4.5, *p* = 0.04).

### Resource densities and resource patch sizes

**Fig. 4:**
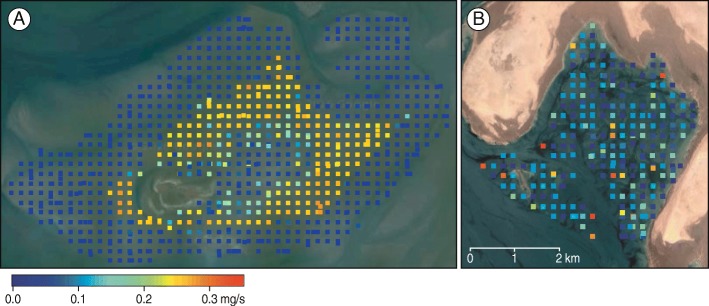
Intake rate by red knots in the Wadden Sea (**a**) and at Banc d’Arguin (**b**) predicted on the basis of estimates of food abundance using grid-sampling. The two maps are to scale, and each square represents one sampling location. The potential intake rate of ash-free dry flesh mass (AFDM_flesh_) was calculated by an experimentally tested diet choice model. Calculations were based on mollusc species making up at least 1% of the red knot’s diet (Table 1). Differences in mean digestive capacity between the tagged Wadden Sea and Banc d’Arguin red knots were considered

**Fig. 5:**
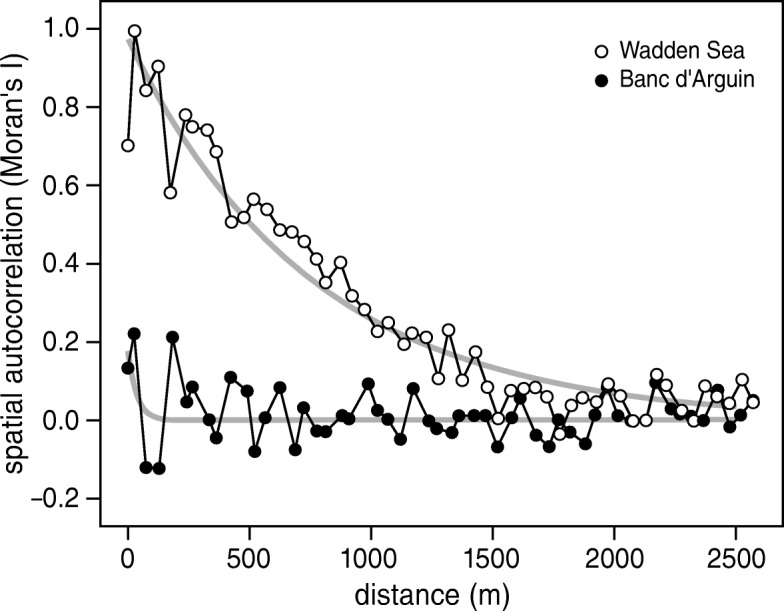
Correlogram of estimated maximum AFDM_flesh_ intake rates in the Wadden Sea and at Banc d’Arguin. Spatial autocorrelation was estimated by calculating Moran’s *I* values, based on the estimated potential AFDM_flesh_ intake rates at the sampling stations (see Fig. 4). Grey lines show exponential regression of the Moran’s *I* values

Maximum resource densities, in terms of available ash-free dry flesh mass per square meter, were much larger in the Wadden Sea (26.2 g AFDM/m^2^) than in Mauritania (7.7 g AFDM/m^2^). Red knots’ intake rate was estimated to be constrained by digestive capacity at 22% of the sampling locations in the Wadden Sea, compared to 4% at Banc d’Arguin. At Banc d’Arguin, the toxin constraint on the intake of *Loripes lucinalis* was estimated to limit intake rate at 48% of the sampling locations. Due to these constraints, the mean potential resource intake rates were similar were similar in the two areas (Fig. 4, mean ± SD, 0.07 ± 0.10 mg AFDM/s in the Wadden Sea and 0.09 ± 0.08 mg AFDM/s on Banc d’Arguin, *p* > 0.1). Also the 95% quantile was similar (0.26 mg AFDM/s in the Wadden Sea, and 0.25 mg AFDM/s on Banc d’Arguin). However, the intercept as well as the range of spatial autocorrelation intercept in the potential resource intake rate was strikingly different between the Wadden Sea (intercept = 0.97, range = 1700 m) and Banc d’Arguin (intercept = 0.18, range < 50 m, Figs 4 and 5, Table 1). Hence, the size of resource patches strongly differed. In the Dutch Wadden Sea, estimated patch size was on average larger (1700 m) than at Banc d’Arguin (50 m, the minimum resolution allowed by the measurements, Fig. 5, Table 1).

**Table 1:** Diet proportion, availability and spatial autocorrelation of molluscs in the Wadden Sea and at Banc d’Arguin

Species^a^	Proportion in diet^b^	Numerical density (1/m^2^)	AFDM_flesh_ (mg/m^2^)	Autocor. intercept	Autocor. range^c^ (m)
Wadden Sea					
*Cerastoderma edule*	0.99	762	760	0.51	900
*Limecola balthica*	< 0.01	31	268	0.04	0
*Peringia ulvae*	< 0.01	427	134	0.52	1600
*Ensis directus*	< 0.01	35	70	0.26	600
*Mya arenaria*	< 0.01	40	38	0.44	1100
*Abra tenuis*	< 0.01	36	34	0.52	700
*AFDM*_*flesh*_ intake rate (mg/s)^d^				0.97	1700
Banc d’Arguin					
*Loripes lucinalis*	0.49	251	1337	0.63	400
*Diplodonta circularis*	0.19	8	101	0.03	0
*Dosinia isocardia*	0.13	25	77	0	0
*Abra tenuis*	0.14	32	31	0.65	0
*Senilia senilia*	0.06	6	29	0.15	200
*AFDM*_*flesh*_ intake rate (mg/s)^d^				0.18	50

^a^Only mollusc species with more than 10 mg AFDM_flesh_ per m^2^ that are in the upper 4 cm of the sediment and ingestible by red knots

^b^AFDM_flesh_ proportion of all listed mollusc prey in the diet, based on dropping data (hinge measurements)

^c^Range is defined as the distance at which the spatial autocorrelation drops below 0.1. The autocorrelation function is estimated by exponential regression of the Moran’s *I* index at discrete distances. Species specific autocorrelation functions are based on the summed AFDM_flesh_ density at each location

^d^Autocorrelation function of the predicted intake rate (see Fig. 5), taking in account only those prey species that had an estimated average proportion of more than 0.01 in the diet

Because resource patches were smaller than the inter-sample distance, the resource sampling scheme at Banc d’Arguin is likely to have missed foraging patches (Figs 4 and 5). Additional sampling of specific locations where tagged red knots were observed foraging showed that they indeed discovered locations where potential energy intake rate was higher than the values obtained for the sampling grid (Additional file 1: Appendix 2, Additional file 1: Figure A1). The analysis of droppings showed that during the tracking period, red knots in the Wadden Sea had a virtually monospecific diet, which for more than 99% of the estimated consumed ash-free dry flesh mass consisted of common cockles, *Cerastoderma edule* (Table 1). At Banc d’Arguin, four different species each contributed more than 10% to the diet (Table 1). In addition, on average 15% of the dropping dry mass at Banc d’Arguin consisted of plant material (ranging between 0 and 85%), presumably rhizomes of seagrass *Zostera noltii*.

## Discussion

Red knots encountered larger resource patches and higher local resource abundances in the Wadden Sea than at Banc d’Arguin. Predicted energy intake rates were similar due to constraints set by digestive capacity. In line with expectations, the differences coincided with stronger aggregation by red knots in the Wadden Sea and stronger individual site fidelity by red knots at Banc d’Arguin. Whereas the Wadden Sea red knots are best described as *grouping nomads* (Fig. 1d), the Banc d’Arguin red knots are at the other side of the space-use spectrum and are best described as *solitary residents* (Fig. 1a). This result suggests that space use patterns do not simply reflect the physical and cognitive traits of red knots; instead, red knots adjust the pattern of space use to the distribution of resources. Below, we discuss alternative explanations for the patterns observed. In particular, we discuss the potential influences of predation risk, individual diet specializations and information use in explaining the observed differences between the areas. But first, we discuss some limitations of the methods.

### From a relative to an absolute measure of aggregation

The here used method does not provide an absolute measure of aggregation. For example, the mean distances between tagged birds (middle boxes in Fig. 3) do not signify inter-individual distances within groups, because they also include the distances between birds in different groups. Also note that the mean distances between tagged birds in the area (y-axis in Fig. 3) must be much larger than the mean distances to other conspecifics in general, because we tracked only a fraction of all red knots in the area. The method used here provides a measure of aggregation that is relative to the total area used by all of the tracked individuals. What we have shown is that red knots in the Wadden Sea generally aggregated in a part of their total used area, whereas the Banc d’Arguin red knots generally spread out across their total used area.

### Limitation of the intake rate model: Unknown food types

Apart from different species of molluscs, at Banc d’Arguin red knot droppings also contained varying amounts of plant material, probably the rhizomes of intertidal seagrass (*Zostera noltii*). Unfortunately, this type of resource cannot be accounted for in the current intake rate models, as energy content of seagrass rhizomes as well as red knots’ search efficiency, handling time, digestive rate and digestion efficiency on this food type have not been measured yet. However, previous studies have argued that seagrass rhizomes contain less nutrients and should be harder to digest by red knots than bivalve flesh [55]. Preliminary experiments confirmed that red knots switch to foraging on seagrass rhizomes only when bivalve densities are very low (JAvG, unpublished data). Hence, the most likely scenario is that red knots only feed on seagrass when bivalve prey is insufficient. Because of this low preference, and the high availability and visibility of seagrass, we expect that it is of minor importance in explaining the space use of red knots. Clearly, further research is needed to confirm this assumption.

### Predation risk

The resource distribution is expected to be the prime determinant of forager movement decisions, as resources are the reason for foragers to move in the first place [75]. As the resource abundance increases, predation risk is expected to become a more important determinant of space use [76]. Indeed, predation risk can be a main determinant of habitat quality for red knots at Banc d’Arguin [50, 77] as well as the Wadden Sea [62]. However, we think that the large differences in space use between Banc d’Arguin and the Wadden Sea are unlikely to be explained by differences in predation risk [75]. Being depredated mainly by falcons, which attack by surprise from behind concealing habitat structures such as ridges of dunes, predation pressure will be relatively low when foraging on the offshore intertidal mudflats [78]. In accordance with that, predation is thought to mainly take place in the two hours before high tide [79], a period that was not included in the analyses. Moreover, even when spaced out, shorebirds maintain the potential to coalesce into tight flocks when necessary [28, 80].

### An information-based approach

Foraging can be viewed as a process by which a forager gathers information on resources. An advantage of foraging in groups is that conspecifics may provide public information [81], which may be more readily available in the large aggregations of red knots in the Wadden Sea. The use of public information by red knots from the Wadden Sea has been shown in an experimental setting [17]. Together with public information, foragers can also make use of personal information from previous experiences. A resource landscape that is stable over time allows foragers to return to suitable foraging locations and thus facilitate site fidelity [6]. If personal experience increases the efficiency of the used foraging strategy, a foraging strategy may become self-reinforcing. Such positive feedback enhances between-individual differences in strategies and could promote solitary foraging. Moreover, several studies have shown that red knots’ digestive physiology adjusts to dietary differences, which may drive foraging decisions even further apart [50, 82]. The analysis of droppings showed that red knots at Banc d’Arguin had a more diverse diet than red knots in the Wadden Sea, who had a virtually monospecific diet of cockles. We did not study dietary differences between individuals, but a part of the diet diversity at Banc d’Arguin may well be the result of different individuals having different diet specializations, as proposed by previous studies [36, 50, 77]. We note, however, that also when feeding in groups on monospecific diets in the Wadden Sea, red knots appear to differ in their individual diet preferences, for example with respect to the range of prey qualities they accept [49, 82].

### The development of space use

We have shown that space use patterns of red knots are not simply a species characteristic, but that they can be explained as a response to the local resource environments. This suggests that when predicting the ecological effects of environmental change, animal space use characteristics should not be assumed to remain stable. Unfortunately, it is difficult to tell what must be assumed instead. If the observed space use differences largely result from genetic differences between the two populations, then adjustment to environmental change would take many generations. Behavioural differences may also result from environmental effects on development, which would imply that adjustment to environmental change can happen in one generation [83, 84]. Even more directly, differences in space use patterns may be explained by direct behavioural responses to the environment, in which case adjustment to changing environments could be instantaneous. The observation that *canutus* red knots blend in with *islandica* red knots when visiting the Wadden Sea during migration [44, 62] suggests that this last possibility is likely to be predominant.

More insight into these processes could be provided by tracking individual red knots as well as their local environment along the different areas that they visit during their annual migratory cycle, or even over multiple years in the same areas. Even if the state of animal tracking technology may restrict the application of such methods, it seems more challenging to track the changes in the resource landscapes experienced by individuals in sufficient spatial and temporal detail. Right now, it may not be the tracking technology that limits progress [85], but the grain size at which we can measure relevant aspects of the environment.

## Conclusions

Foraging red knots in the Wadden Sea showed a high degree of aggregation, whereas foraging red knots at Banc d’Arguin did not. In contrast, red knots in the Wadden Sea did not show site fidelity, which the Banc d’Arguin red knots did. These contrasting strategies (grouping nomads versus solitary residents) fit with the observed differences in the resource landscapes. The single prey species in the Wadden Sea occurred in large patches, which allowed red knots to forage in large groups. Contrarily, the multiple prey species at Banc d’Arguin occurred scattered in small patches, which increases interference competition but may allow foragers to monopolize a patch and postpone depletion. We conclude that space use patterns by red knots are not simply a species characteristic, but can be explained as a response to the local resource environments.

## Additional file

Additional file 1: Supplementary material. (PDF 430 kb)

## Abbreviations

AFDM_flesh_: Ash-free dry flesh mass
DM_shell_: Dry shell mass
