# Publisher Correction: Resource landscapes explain contrasting patterns of aggregation and site fidelity by red knots at two wintering sites

**DOI:** 10.1186/s40462-019-0151-y

**Published:** 2019-02-21

**Authors:** Thomas Oudman, Theunis Piersma, Mohamed V. Ahmedou Salem, Marieke E. Feis, Anne Dekinga, Sander Holthuijsen, Job ten Horn, Jan A. van Gils, Allert I. Bijleveld

**Affiliations:** 1 NIOZ Royal Netherlands Institute for Sea Research, Department of Coastal Systems, and Utrecht University, P.O. Box 59, 1790 AB Den Burg, Texel The Netherlands; 2 Centre for Biological Diversity, School of Biology, University of St Andrews, Fife, KY16 9TF UK; 3 Rudi Drent Chair in Global Flyway Ecology, Conservation Ecology Group, Groningen Institute for Evolutionary Life Sciences (GELIFES), University of Groningen, PO Box 11103, 9700 CC Groningen, The Netherlands; 4 EBIOME Ecobiologie Marine et Environnement, Département de Biologie, L’université de Nouakchott Al-Aasriya, BP 880 Nouakchott, Mauritania; 5 Present Address: Sorbonne Université, CNRS, Station Biologique de Roscoff, Laboratoire Adaptation et Diversité en Milieu Marin, UMR 7144, CS90074, 29688 Roscoff Cedex, France

**Correction to: Movement Ecology (2018) 6:24**

**https://doi.org/10.1186/s40462-018-0142-4**

In the original publication of this article [1], the majority of the authors’ proof corrections weren’t processed due to a typesetting mistake. The original publication of this article has been updated to correct the missed corrections. An overview of the corrections can be found in Additional file 1.

The publisher apologises to the readers and authors for the inconvenience.

## Additional file

Additional file 1: Overview of authors’ proof corrections. (DOCX 27 kb)

## References

[1] Oudman, et al. Resource landscapes explain contrasting patterns of aggregation and site fidelity by red knots at two wintering sites. Movement Ecology. 2018;6(24). 10.1186/s40462-018-0142-4. 10.1186/s40462-018-0142-4 PMC6300905 30598823

