# Supplementary material for: Resource landscapes explain contrasting patterns of aggregation and site fidelity by red knots at two wintering sites
Source: Mov Ecol. 2018 Dec 20;6:24. doi: 10.1186/s40462-018-0142-4 (PMC6300905; doi:10.1186/s40462-018-0142-4)
Supplement: Supplementary file 1 — Supplementary material. (PDF 430 kb) [file 40462_2018_142_MOESM1_ESM.pdf]

APPENDIX 1. Outline of the functional response model

The functional response model that we used in this study is generally referred to as the Toxin-Digestive Rate Model (TDRM) [1], and was developed for red knots that forage under non-ad libitum circumstances and need to search for their prey. The full model is outlined in [1]. Below, we provide a concise explanation of the model, and a detailed explanation of its current parameterization.

The model is based on the idea of a forager searching for prey in an environment with different prey types of limited availability. The goal of the model is to estimate for each prey type the optimal acceptance probability (p_i) such that long-term energy intake rate (Y) is maximized, and to calculate this rate.

To do so, each prey type is assumed to have a specific energy content (e_i, estimated for molluscs as the ash-free dry flesh mass, $AFDM_{flesh}$), and a specific ballast mass (k_i, estimated for molluscs as the dry shell mass, DM_{shell}). It is further assumed that the forager has a maximum ballast mass intake rate, referred to as digestive capacity (c, [mg DM_{shell}/s]). Digestive capacity varies among individual red knots and scales to the square of gizzard mass [2, 3]. Gizzard sizes were measured non-invasively by ultrasonography [4, 5] immediately after the catch by AD. Gizzard mass was estimated from the observed sizes as described in [4] and were lower in the Wadden Sea (mean ± SD, 7.0 ± 2.0 g) than at Banc d'Arguin (8.5 ± 1.8 g). From these values, the digestive constraint c was estimated using an experimentally derived calibration curve [6] as 2.5 mg DM_{shell}/s in the Wadden Sea and 3.7 mg DM_{shell}/s at Banc d'Arguin. For one of the prey species that is absent from the Wadden Sea but abundant in Banc d'Arguin, *Loripes lucinalis*, the maximum intake rate is not set by digestive rate, but by its toxicity due to a high sulfur content

[7]. This toxin constraint is denoted by q and has been experimentally derived as 0.1 mg $AFDM_{flesh}$/s [6, 7].

Given the density (D_i, [number/m^2]), handling time (h_i, [s]), $AFDM_{flesh}$ mass (e_i, [g]) and DM_{shell} mass (k_i, [g]) of each prey type, the model uses a graphical procedure to derive the optimal combination of acceptance probabilities (p_i) for all available prey types, such that the $AFDM_{flesh}$ intake rate Y (mg $AFDM_{flesh}$/s) is maximized, but without surpassing the digestive constraint c and the toxin constraint q. For details of the optimization procedure, we refer to [1]. Y is derived from Hollings disc equation for multiple prey types [8], and is calculated as:

$$Y = \frac{\sum_i p_i a D_i e_i}{1 + \sum_i p_i a D_i h_i}. \qquad (eq. 1)$$

a is the searching efficiency for each prey type i, estimated at 6.4 cm^2/s in the Wadden Sea [9] and, due to obstruction by seagrass roots, at 2.0 cm^2/s at Banc d'Arguin [10]. Similarly, ballast mass intake rate, which must remain below the digestive constraint, was calculated as:

$$c > \frac{\sum_i p_i a D_i k_i}{1 + \sum_i p_i a D_i h_i}. \qquad (eq. 2)$$

The toxin constraint was defined as

$$q > \frac{p_l a D_l e_l}{1 + \sum_i p_i a D_i h_i}, \qquad (eq. 3)$$

where p_l, D_l and e_l are the prey-type specific values for *Loripes lucinalis*.

Handling time of each prey type (h_i) was assumed be a function of shell size, previously estimated for *Cerastoderma edule* as 3.3 x length [cm] ^{2} [9]. *Senilia senilis* in Banc d'Arguin is similarly shaped, and therefore the same estimates were used. The other relevant molluscs at Banc d'Arguin (*Loripes lucinalis*, *Dosinia isocardia*, *Diplodonta circularis* and *Abra tenuis*) were assumed to have handling times similar to *Limecola balthica* (2.1 x length^2 when buried at an average depth of 2 cm) [9], as they are all relatively flat and round burying bivalves with a

smooth surface. To estimate $AFDM_{flesh}$ mass (e_i) and DM_{shell} mass (k_i) of each prey, the flesh and shell were separated of around one hundred individuals of each prey species, and then dried at 60 °C for 3 days and weighed (mg). Flesh was then incinerated for 5 h at 560 °C and weighed again to determine $AFDM_{flesh}$. By linear regression on log-transformed values, $AFDM_{flesh}$ and DM_{shell} were estimated as a function of shell length for each species separately [2, 11]. All parameters used in the model and their descriptions are given in Table A1.

Table A1. Parameters used in the functional response model

Parameter	Value	Unit	Description	Reference
a_w	6.4	cm^2/s	Search efficiency Wadden Sea	[9]
a_b	2.0	cm^2/s	Search efficiency Banc d'Arguin	[10]
c_w	2.5	mg DM_{shell}/s*	Digestive constraint Wadden Sea	[4], this study
c_b	3.7	mg DM_{shell}/s	Digestive constraint Banc d'Arguin	[4], this study
q	0.1	mg $AFDM_{flesh}/s$*	Toxin constraint on *Loripes lucinalis* intake	[7]
h_i	al^2	s	Handling time, where l is shell length in cm. a is 3.3 for *Cerastoderma edule* and *Senilia senilis* and 2.1 for the other species.	[9]
e_i	$10^{b+c\,log(l)}$	mg $AFDM_{flesh}$	Energy content per prey item, where l is shell length in cm. b and c were fitted by species specific calibration curves.	[11], this study
k_i	$10^{d+e\,log(l)}$	mg DM_{shell}	Ballast mass per prey item, where l is shell length in cm. d and e were fitted by species specific calibration curves	[11], this study

*DM_{shell} stands for dry shell mass, and $AFDM_{flesh}$ stands for ash-free dry flesh mass

APPENDIX 2. Additional sampling at foraging locations at Banc d'Arguin

The estimated range of resource patches in the Banc d'Arguin, 50 m, was smaller than the inter-sampling distance of 250 m. Given the low autocorrelation intercept (*Moran's I* = 0.18), resource patches may have been smaller than the sampling accuracy, approximately 10 m. Therefore, it is expected that many resource patches were actually missed by the sampling grid. To verify this, we additionally performed an alternative sampling scheme, based on the idea that red knots are the champions when it comes to finding resource patches. Sampling locations were determined in the field. Two observers with telescopes searched for tagged red knots in the field, careful not to disturb foraging flocks of red knots. When a tagged red knot was observed, usually from a distance of 150-250 m, the knot and its precise location was carefully observed. After the red knot flew away, one of the observers guided the other observer to the exact foraging location, without losing sight of the location through the telescope. The location was stored in a GPS, and eight wooden picks were placed at foraging traces (holes left by a red knot bill, droppings, or footprints). A sample was taken at each of the picks within the following week, according to the same protocol as described in the main text, but on foot during low tide rather than by boat during high tide.

Given that red knots need an average energy intake of 0.2 mg $AFDM_{flesh}$ s^{-1} to maintain a stable body mass in Banc d'Arguin [12], only 7% of the locations in the sampling grid in the Banc d'Arguin harboured enough mollusc biomass (Fig. A2b). Contrarily, at 70% (34 out of 44) of the locations where tagged birds were observed foraging, at least one sample surpassed this threshold (Fig. A1). This way of sampling uncovered many more resource patches than the grid (compare Fig. A1 with Fig. 4b in main text).

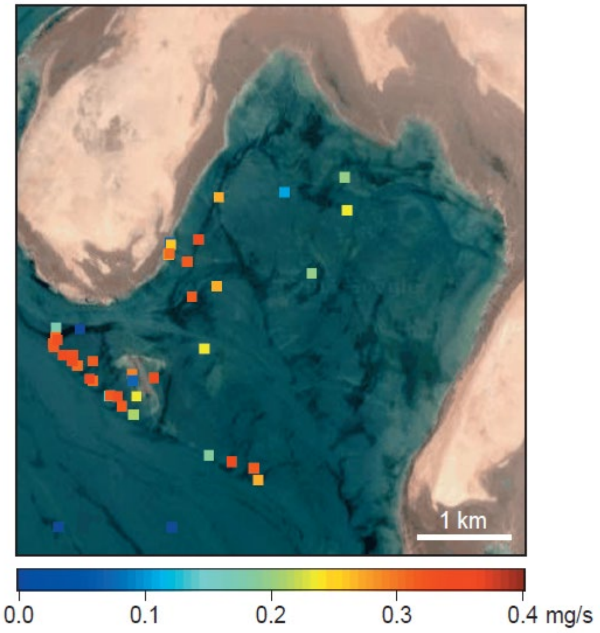

Figure A1. Potential intake rates by red knots at additional sampling locations at Banc d'Arguin. See Appendix 1 for explanation of this additional sampling scheme. Each square refers to one location, and the colour refers to the potential intake rate (mg $AFDM_{flesh}$ /s) at those locations. Samples with higher intake rates are plotted on top of samples with lower intake rates. Spatial scale and colour scale are the same as in Fig. 4 in the main text.

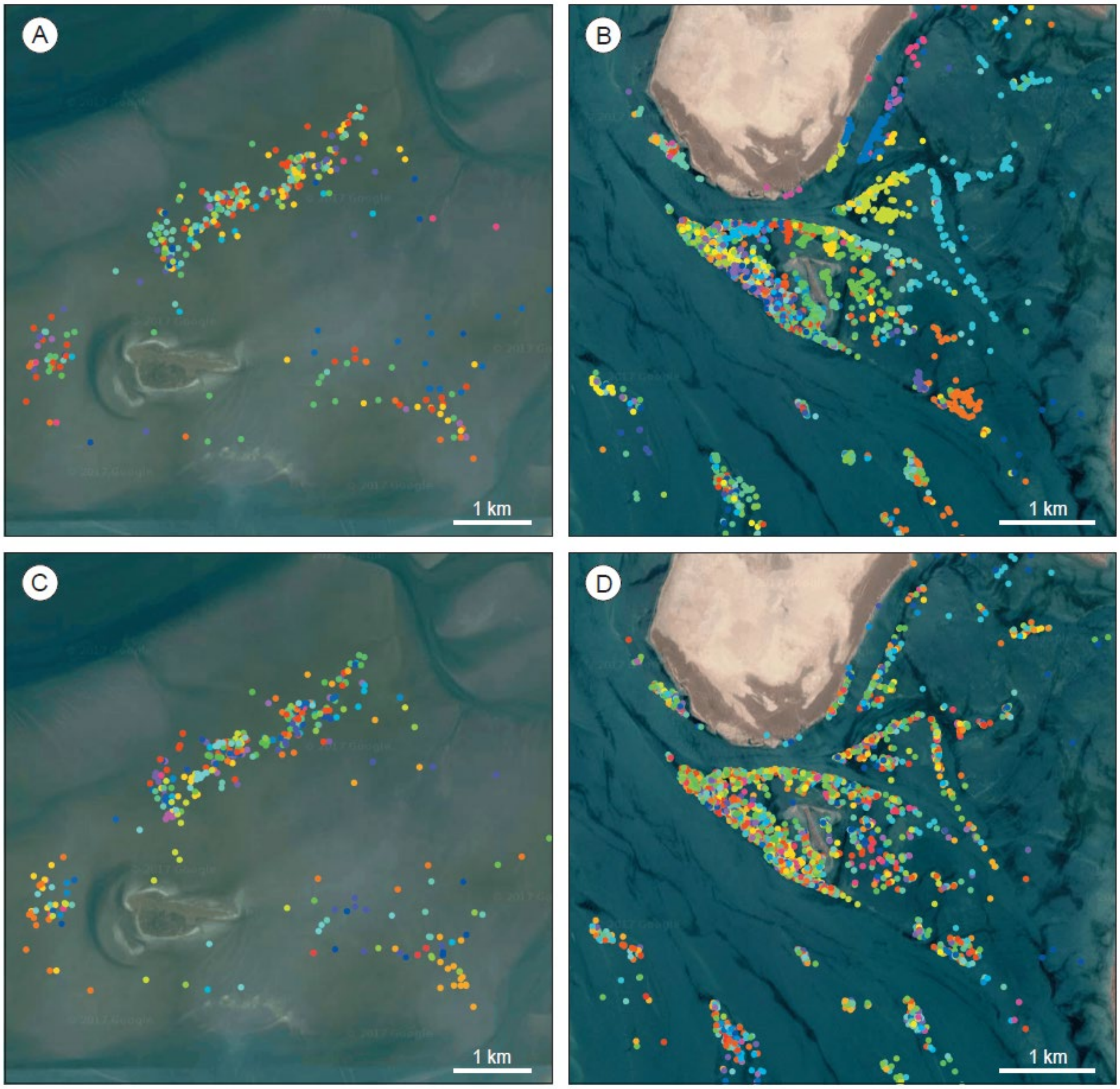

Figure A2. Map of residence patches of tracked red knots in the Dutch Wadden Sea (a and c) and at Banc d'Arguin (b and d). Each dot denotes one residence patch. In panels a and b, each color denotes a different individual. Panels b and d show the same residence patches, but now each color refers to a single low tide period. Note that the spatial scale slightly differs between the left and the right panels.

ADDITIONAL INFORMATION REFERENCES

1. van Gils JA, van der Geest M, Leyrer J, Oudman T, Lok T, Onrust J, et al. Toxin constraint explains diet choice, survival and population dynamics in a molluscivore shorebird. Proc R Soc B Biol Sci 2013;280:20130861. (doi:10.1098/rspb.2013.0861).
2. van Gils JA, de Rooij SR, van Belle J, van der Meer J, Dekinga A, Piersma T, et al. Digestive bottleneck affects foraging decisions in red knots *Calidris canutus*. I. Prey choice. J Anim Ecol 2005;74:105-19. (doi:10.1111/j.1365-2656.2004.00903.x).
3. van Gils JA, Piersma T, Dekinga A, Dietz MW. Cost-benefit analysis of mollusc-eating in a shorebird II. Optimizing gizzard size in the face of seasonal demands. J Exp Biol 2003;206:3369-80. (doi:10.1242/Jeb.00546).
4. Dietz MW, Dekinga A, Piersma T, Verhulst S. Estimating organ size in small migrating shorebirds with ultrasonography: an intercalibration exercise. Physiol Biochem Zool 1999;72:28-37.
5. Dekinga A, Dietz MW, Koolhaas A, Piersma T. Time course and reversibility of changes in the gizzards of red knots alternately eating hard and soft food. J Exp Biol 2001;204:2167-73.
6. Oudman T, Hin V, Dekinga A, van Gils JA. The effect of digestive capacity on the intake rate of toxic and non-toxic prey in an ecological context. Plos One 2015;10:e0136144. (doi:10.1371/journal.pone.0136144).
7. Oudman T, Onrust J, de Fouw J, Spaans B, Piersma T, van Gils JA. Digestive capacity and toxicity cause mixed diets in red knots that maximize intake rate. Am Nat 2014;183:650-9.
8. Holling CS. Some characteristics of simple types of predation and parasitism. Can Entomol 1959;91:385-98. (doi:10.4039/Ent91385-7).
9. Piersma T, van Gils JA, de Goeij P, van der Meer J. Holling's functional-response model as a tool to link the food-finding mechanism of a probing shorebirds with its spatial distribution. J Anim Ecol 1995;64:493-504. (doi:10.2307/5652).
10. de Fouw J, van der Heide T, Oudman T, Maas LRM, Piersma T, van Gils JA. Structurally complex seagrass obstructs the sixth sense of a specialized avian molluscivore. Anim Behav 2016;115:55-67.
11. Oudman T, Bijleveld AI, Kavelaars MM, Dekinga A, Cluderay J, Piersma T, et al. Diet preferences as the cause of individual differences rather than the consequence. J Anim Ecol 2016;85:1378-88. (doi:10.1111/1365-2656.12549).
12. van Gils JA, van der Geest M, Kraan C, Folmer EO, Jansen EJ, Piersma T. How the carrying capacity of the Wadden Sea regulates the number of wintering waders at Banc d'Arguin. Limosa 2009;82:134-40.
