# Supplementary material for: Publisher Correction: Resource landscapes explain contrasting patterns of aggregation and site fidelity by red knots at two wintering sites
Source: Mov Ecol. 2019 Feb 21;7:5. doi: 10.1186/s40462-019-0151-y (PMC6385434; doi:10.1186/s40462-019-0151-y)
Supplement: Supplementary file 1 — Overview of authors’ proof corrections. (DOCX 27 kb) [file 40462_2019_151_MOESM1_ESM.docx]

| **Section** | **Paragraph** | **Correction** |
| --- | --- | --- |
| Abstract | 3rd | "space use pattern" replaced by "space use" |
| Caption Figure 1 | - | "in population" replaced by "of" |
| Caption Figure 1 | - | ", which" replaced by "that" |
| Measuring aggregation, site fidelity, and the resource landscape in two contrasting environments | 2nd | “to the data from the resource-sampling scheme” replaced by "to data from resource sampling." |
| Measuring aggregation, site fidelity, and the resource landscape in two contrasting environments | 2nd | “1 %” replaced by” 1%” |
| Measuring aggregation, site fidelity, and the resource landscape in two contrasting environments | 3rd | Reference citation “[40]” moved to a different position within the sentence |
| Methods | 2nd | “At Banc d’Arguin, 46 red knots were released with a tag, and tracked between 9 January and 13 February 2013 [50].” replaced by ” "From a similar study at Banc d’Arguin [50], we used data from 38 red knots that were tracked between 9 January and 13 February 2013." |
| Measuring aggregation and site fidelity | 1st | 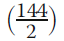replaced by 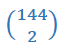  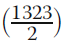 replaced by 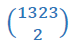 |
| Caption Figure 2 |  | Added at the end of caption of Fig. 2 “ . Insets below the x-axis are visual aids: the dotted lines represent the distance measures summarized in the boxes above. Symbols correspond to Fig. 1" |
| Measuring aggregation and site fidelity | 1st | "non-randomized and the randomized distances." replaced by "categorized mean distances". |
| Resource sampling | 2nd | "In" replaced by "With small" |
| Resource sampling | 2nd | "shell" by "flesh" |
| Aggregation and site fidelity of tagged red knots | 1st | “"grouping nomads" " replaced by " *grouping nomads "*  (i.e. without quotation marks but in italics) |
| Aggregation and site fidelity of tagged red knots | 2nd | "p < 0.01" replaced by "p < 0.001" |
| Calculating potential resource intake rates | 1st | "prey." Replaced by "prey [33]." |
| Aggregation and site fidelity of tagged red knots | 2nd | " "solitary residents" “ replaced by " *solitary residents* "  (i.e. without quotation marks but in italics) |
| Resource densities and resource patch sizes | 1st | "were similar were similar" replaced by "were similar" |
| Resource densities and resource patch sizes | 1st | removed "intercept" |
| Caption Figure 3 |  | Added at the end of caption of Fig. 3 “Insets below the x-axis are visual aids, as in Fig. 2: the dotted lines represent the distance measures summarized in the boxes above. In the bird-symbols, each color represents one individual" |
| Resource densities and resource patch sizes | 2nd | Added after "diet": ", and 95% all together" |
| Table 1 |  | Aligned all numbers to the right instead of the left |
| Predation risk | 1st | "increases" replaced by "becomes less limiting" |
| From a relative to an absolute measure of aggregation | 1st | "The here used method does" replaced by "Our method did" |
| From a relative to an absolute measure of aggregation | 1st | "The method used here provides" replaced by  "Our method provided" |
| References | 1 | Replaced "theory by "Theory" |
|  | 2 | Added "https://doi.org/10.1007/BF01601953." |
|  | 6 | Added "https://doi.org/10.1007/BF01237820." |
|  | 12 | "2017." replaced by "2017;126:1004–19." |
|  | 13 | Removed brackets around the DOI-link |
|  | 15 | Added "https://doi.org/10.1139/z90-092." |
|  | 18 | Added "https://doi.org/10.5253/arde.v68.p31." |
|  | 19 | Added "https://doi.org/10.1093/beheco/ari067." |
|  | 20 | "ecology: an evolutionary approach" replaced by  "Ecology: an Evolutionary Approach" |
|  | 21 | Added "https://doi.org/10.1111/1365-2656.12309." |
|  | 25 | Added "https://www.jstor.org/stable/4159278." |
|  | 26 | Added "https://www.jstor.org/stable/4159278" |
|  | 27 | Added "https://www.jstor.org/stable/2096773." |
|  | 31 | "FMD M" replaced by ""Marquitti FMD" |
|  | 32 | Added "https://doi.org/10.1111/j.1600-0706.2013.00406.x." |
|  | 35 | Added "https://doi.org/10.1086/675759." |
|  | 37 | "JGC H" replaced by "Hopcraft JGC" |
|  | 39 | "CJA B" replaced by "Bradshaw CJA" |
|  | 39 | (http://www.nature.com/nature/journal/v451/n7182/suppinfo/  nature06518_S1.html) replaced by <https://doi.org/10.1038/nature06518.> and removed brackets around the link. |
|  | 43 | Hyperlinked “https://  doi.org/10.1650/0010-5422(2005)107[0497:pdtahd]2.0.co;2.” |
|  | 44 | "88:11." Replaced by "88:65–76." |
|  | 45 | Hyperlinked https://doi.org/10.1890/0012-9658(2006)87[1189:fiatse]2.0.co;2. |
|  | 48 | "RB MC" replaced by "MacCurdy RB" |
|  | 49 | "RB MC" replaced by "MacCurdy RB" |
|  | 49 | Added "https://doi.org/10.1098/rspb.2015.1557." |
|  | 53 | "handbook of position location: theory, practice, and advances"  Replaced by "Handbook of Position Location: Theory, Practice, and Advances" |
|  | 55 | Added "https://doi.org/10.1126/science.aad6351." |
|  | 56 | "LRM L" replaced by "Maas LRM" |
|  | 56 | Added "https://doi.org/10.1016/j.anbehav.2016.02.017." |
|  | 58 | "1–15" replaced by " "197–211" |
|  | 59 | "273" replaced by "20160253" |
|  | 62 | Added "https://doi.org/10.1016/0077-7579(93)90052-T." |
|  | 65 | Added "https://doi.org/10.1890/14-1845.1." |
|  | 66 | Added "https://doi.org/10.1080/00063659309477140." |
|  | 70 | Added "https://doi.org/10.1086/316648." |
|  | 73 | Added "https://doi.org/10.3354/meps07994." |
|  | 75 | "birds and habitat: relationships in changing landscapes" replaced by "Birds and Habitat: Relationships in Changing Landscapes"  "fuller R)." replaced by "Fuller R), pp 383–407." |
|  | 79 | "banc" replaced by "Banc" |
|  | 80 | "LRM L" replaced by "Maas LRM" |
|  | 81 | Added "https://doi.org/10.1098/rstb.2002.1064." |
|  | 83 | "Developmental plasticity and evolution" replaced by  "Developmental Plasticity and Evolution" |
|  | 84 | "The flexible phenotype: a body-centred integration of ecology, physiology and behaviour" replaced by  "The Flexible Phenotype: a Body-Centred Integration of Ecology, Physiology and Behaviour" |
|  | 85 | Added "https://doi.org/10.1126/science.aaa2478." |
